# Chaotic systems with variable indexs for image encryption application

**DOI:** 10.1038/s41598-022-24142-4

**Published:** 2022-11-15

**Authors:** Minxiu Yan, Jingfeng Jie, Ping Zhang

**Affiliations:** School of Information Engineering, Shenyang University of Chemical Technology, Shenyang, 110142 China

**Keywords:** Mathematics and computing, Physics

## Abstract

A new chaotic system is obtained by changing the number of unknown parameters. The dynamical behavior of the chaotic system is investigated by the exponential change of the single unknown parameter and the state variable in the nonlinear term of the system. The structure of the newly constructed chaotic system is explored. When the number of the same state variables in the nonlinear term of the chaotic system varies, the system’s dynamic behavior undergoes complex changes. Moreover, with the exponential change of a single-state variable in a three-dimensional system, the system maintains the chaotic attractor while the state of the attractor changes. On this basis, the Lyapunov exponent, bifurcation diagram, complexity, and 0–1 test are used to compare and analyze this phenomenon. Through circuit simulations, the chaotic characteristics of the system under different conditions are further verified; this provides a theoretical basis for the hardware implementation of the new system. Finally, the new chaotic system is applied to an image encryption system with the same encryption and decryption processes. The comparison shows improved encryption and decryption characteristics of image encryption systems.

## Introduction

Since meteorologist Edward Lorenz discovered the first chaotic attractor, research on chaos has entered a period of rapid development. Many scholars have begun to organically combine chaotic systems with other fields to promote the study and application of chaotic systems among interdisciplinary studies. For example, the chaotic system is applied to secure communication, image encryption, environmental pollution prevention, and soil salinization analysis, among many other fields^1–8^. For the interdisciplinary research of chaotic systems, the chaotic system model is the basis for development and application. In recent years, chaotic systems with a single scroll, multiple scroll, single scroll coexistence, multiple scroll coexistence, or infinite scroll, have been developed^9–13^. It is common to obtain appropriate chaotic systems based on previous studies. The improved systems are mainly divided into self-excited attractors and hidden attractors^14–16^. Developing a new chaotic system with rich dynamic characteristics is particularly important for practical engineering applications^17–19^.

Some studies have shown that chaotic systems can enable systems to exhibit chaotic properties by varying the structure of the chaotic system model, such as using memristors or mathematical functions^20–24^. Liu et al.^25^ added an exponential function to a chaotic system with no equilibrium point. The new design shows richer dynamic characteristics and successfully applies the system to hardware circuit implementations. Yan et al.^26^ applied memristive characteristics to a system model to allow the chaotic system to adjust the system model under different conditions to further deepen the uncertainty of the system. Sun et al.^27^ introduced a tangent function to a chaotic system to realize the self-replication of the chaotic attractor. Intermittent chaos and infinite countable and uncountable attractors coexist, and the system is successfully applied to image encryption. Zhou et al.^28^ used a nonlinear term that includes a sine function and successfully observed the coexistence of multiple attractors in a system under specific initial conditions. The changes in chaotic systems are greatly affected by unknown parameters. Abdulaziz et al.^29^ improved the new chaotic system model by controlling the selection of unknown parameters. The dependency of the generation of chaotic attractors on the parameters affects the system’s comlpexity and has a positive effect on the Lyapunov exponent. Yan et al.^30^ designed a simple three-dimensional chaotic system with unique variable parameters, and demonstrated the complex dynamics of the system by varying the parameters values. By introducing the parameter selection mechanism of the chaotic system. Zheng et al.^31^ modified the introduced state variables and cipher images, dynamically changing the parameters of the disturbance Logistic map. Therefore, the system has good anti-attack capability. Wang et al.^32^ proved through theoretical analysis that the chaotic system could resist dynamic degradation. Kengne et al.^33^ designed a new adaptive chaotic oscillator with a pair of antiparallel semiconductor diodes. The system has disconnected attractor coexistence. Tsafack et al.^34^ proposed a RLC oscillator circuit with chaotic memory and applied the system to image encryption. The results are verified by standard image security analysis techniques. Njitacke et al.^35^ studied bidirectionally coupled neurons, exploring their equilibrium and stability. Extraordinary phenomena with chaos were discovered, such as chaotic peaks at rest. On this basis, a new wide-range chaotic system-coupled map lattice model with one-dimensional and two-dimensional parameters was developed and applied to the newly proposed image encryption algorithm.

Nowadays, scholars mainly focus on exploring the chaotic characteristics generated by chaotic systems. For example, whether a variety of chaotic attractors can be generated, whether different dynamic behaviors can be generated by varying switching terms, and so on. Few studies investigate the effect of unknown parameters on the dynamic characteristics of chaotic systems. In particular, the effect of the number of unknown parameters with the same value on the dynamic behavior of the system has rarely been mentioned in literature. Moreover few studies exist on the dynamic behavior changes in the same chaotic system due to the change in the nonlinear terms.

In addition, some significant results are obtained by applying the chaotic properties of chaotic systems to image encryption systems. For example, scholars have used the chaotic IWT-LSB blind watermarking method with flexible capacity to safely transmit medical images^36^, or proposed a new blind watermarking scheme for medical images based on Schur triangulation and chaotic sequences^37^. In a wireless communication scheme implemented with a PIC microcontroller on the Zigbee channel^38^, chaotic mapping is used to improve the randomness of image encryption. Alternatively, the enhanced sequence of a chaos map is used to encrypt real-time RGB images for IoT applications^39^. Nevertheless, many potential application cases still exist, necessitating further deepening of the strudy of chaotic systems . In this study, a newly proposed chaotic system is applied to an image encryption system and the related encryption and decryption properties are explored; this provides a theoretical basis for the application of the chaotic system with a variable number of unknown parameters to image encryption.

Now, the structure of the classical Lorenz system is modified to obtain a chaotic system with the same value of unknown parameters and a variable number of parameters. Meanwhile, the exponential of the single-state variable of the nonlinear term changes numerically in the system structure of the new system. This paper presents a detailed analysis of the numerical changes of the state variable index of the new system and the dynamic behavior changes of the chaotic systems. The new chaotic system is successfully applied to circuit simulations as well as to a chaotic image encryption system. To verify the feasibility of the new chaotic system in practical applications, the related encryption characteristics of the new system are compared with those in other chaotic image encryption cases.

The paper is mainly structured as follows. In sections "New system model", "Equilibrium points of the new system", and "Effect of variable parameters on the dynamic behavior of the new system", the dynamic behavior of the new improved chaotic system is expounded, including the sensitivity to the initial values of the parameters and the characteristics of the equilibrium point. Section "The effect of the number of unknown parameters on the system dynamic behavior" describes the change in the chaotic characteristics of the new chaotic system when the number of unknown parameters with the same value varies; it then illustrates the similarities and differences of the new chaotic system‘s dynamic behavior with examples. In section "Exponential variation characteristics of nonlinear terms of the new system", the dynamic behavior changes caused by the exponential changes in nonlinear term of single-state variables in the new system are analyzed by examples. The bifurcation diagram and 0–1 test are used to compare and analyze the related behaviors. Section "Circuit simulation analysis" analyzes the circuit simulations of the new chaotic system under different initial conditions, and explains the simulation results. In Sections "Image encryption processing" and "Simulation results and performance analysis", the application of the new chaotic system to an image encryption system is presented, the application steps are introduced, and the encryption and decryption effects are analyzed by analogy.

## New system model

In 2008, Sun et al.^40^ proposed a simplified Lorenz system. The system is controlled by a single parameter and contains a three-parameter dynamic behavior. The simplified system model is :

1 $$\begin{aligned} {\left\{ \begin{array}{lll} {\dot{x}}=10(y-x)\\ {\dot{x}}=-xy+(24-4c)x+cy\\ {\dot{x}}=xy-z8/3\\ \end{array} \right. } \end{aligned}$$

By changing the nonlinear terms in the simplified chaotic system (1), a chaotic system controlled by a single unknown parameter is obtained. The system model is:

2 $$\begin{aligned} \left\{ \begin{array}{lll} {\dot{x}}_{1}=-ax_1+x_2x_3\\ {\dot{x}}_{2}=ax_1-x_1x_3\\ {\dot{x}}_{3}=-ax_3+x_1^2+a\\ \end{array} \right. \end{aligned}$$

where $x_1$, $x_2$, and $x_3$ are state variables, and the system contains a single unknown parameter *a* ($a > 0$). The system has seven terms, including a constant term. The system’s structure is relatively simple, but it has complex dynamics.

**Table 1:** Comparison of structural features with other systems.

System model	Structural characteristic	References
$$\left\{ \begin{array}{lll} {\dot{x}}=y-z \\ {\dot{y}}=ay-x^2z \\ {\dot{z}}=-z+x\\ \end{array} \right. $$	Unadjustable number of control parameters, single parameter range	Ref.^30^
$$\left\{ \begin{array}{lll} {\dot{x}}=y \\ {\dot{y}}=-axz \\ {\dot{z}}=y-bz+y^2-yz\\ \end{array} \right. $$	Multi-control parameters, with strong parameter constraints	Ref.^41^
$$\left\{ \begin{array}{llll} {\dot{x}}=-ay \\ {\dot{y}}=bwz+d \\ {\dot{z}}=y^2-cz^2+e\\ {\dot{w}}=x+y-wz-yz\\ \end{array} \right. $$	Multi-parameter control, harsh parameter constraints, the system contains multiple nonlinear terms	Ref.^42^
$$\left\{ \begin{array}{llll} {\dot{x}}_{1}=-ax_1+x_2x_3 \\ {\dot{x}}_{2}=ax_1-x_1x_3 \\ {\dot{x}}_{3}=-ax_3+x^2_1+a\\ \end{array} \right. $$	Adjustable number of parameters, simple system structure, diverse parameter range	This paper

Through the analysis of Table 1, compared with the system models of chaotic systems in other literature, the advantages of the system model constructed in this paper in terms of control parameters and system structure can be observed.

**Figure 1:**
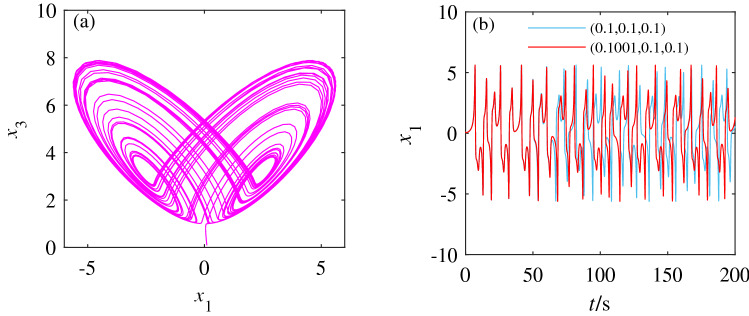
Attractor diagram and time series diagram. (**a**) Parameter $a = 3$, the attractor diagram of $x_1-x_3$ plane when the initial value is (0.1, 0.1, 0.1). (**b**) Time series comparison diagram with initial values of (0.1, 0.1, 0.1) and (0.1001, 0.1, 0.1).

The unknown parameter *a* is assigned a value of $a = 3$ and the initial value is selected as (0.1,0.1,0.1). The chaotic attractor diagram of system (2) is shown in Fig. 1a. Meanwhile, to study the sensitivity of system (2) to the initial value, we compare the sequence diagrams with the initial value (0.1001,0.1,0.1) and with the initial value (0.1,0.1,0.1). Figure 1b shows the effect of small changes in initial values on the dynamic behavior of system (2). The chaotic characteristics of system (2) have obviously changed, indicating that system (2) is susceptible to the initial value.

## Equilibrium points of the new system

The left end of the equation for system (2) is assigned to:

3 $$\begin{aligned} \left\{ \begin{array}{lll} 0=-ax_1+x_2x_3\\ 0=ax_1-x_1x_3\\ 0=-ax_3+x_1^2+a\\ \end{array} \right. \end{aligned}$$

The equilibrium points of the system: $E_1=(0,0,1)$, $E_2=(-\sqrt{a(a-1)},-\sqrt{a(a-1)},a)$, and $E_3=(\sqrt{a(a-1)},\sqrt{a(a-1)},a)$.

Because system (2) has chaotic attractor when parameter $a = 3$, the selected parameter value is substituted at the equilibrium point $E_1=(0, 0, 1)$, the Jacobian matrix *J* of the system can be obtained:

4 $$\begin{aligned} J = \left[ \begin{array}{ccc} -a &{} x_3 &{} x_2 \\ a-x_3 &{} 0 &{} -x_1 \\ 2x_1 &{} 0 &{} -a \end{array} \right] = \left[ \begin{array}{ccc} -3 &{} x_3 &{} x_2 \\ 3-x_3 &{} 0 &{} -x_1 \\ 2x_1 &{} 0 &{} -3 \end{array} \right] \end{aligned}$$

The eigenvalues of the corresponding Jacobian matrix should satisfy the following equation:

5 $$\begin{aligned} f(\lambda )={\lambda }^3+A_2{\lambda }^2+A_1\lambda +A_0 \end{aligned}$$

where $$A_2=15x_3-5x_3^2$$, $$A_1=15-2x_1x_2+3x_3-x_3^2$$, and $$A_0=8$$.

According to the Routh-Hurwitz criterion, the system has a stable equilibrium point when $A_0>0,A_2>0,$ and $A_2A_1-A_0>0$. The corresponding characteristic roots and the types of equilibrium points are obtained as shown in Table 2.

**Table 2:** Equilibrium points and stability of the system.

Equilibrium points	Latent roots	Stability judgment
$E_1$	$\lambda _1$=0.5616, $\lambda _2$=− 3.0, $\lambda _3$=− 3.562;	Unstable
$E_2$	$\lambda _1$=− 7.1291, $\lambda _2$=0.56456+2.1751i, $\lambda _3$=0.56456 − 2.1751i;	Unstable
$E_3$	$\lambda _1$=− 7.129, $\lambda _2$=0.56456+2.1751i, $\lambda _3$=0.56456 − 2.1751i;	Unstable

Table 2 shows that the characteristic root obtained by substituting the equilibrium point $E_1$ into the characteristic equation contains one positive real root and two negative real roots, indicating that the equilibrium point $E_1$ is a saddle-focus equilibrium point with index 1^43^. Substituting the equilibrium points $E_2$ and $E_3$ into the characteristic equation, the characteristic root contains a negative real root and a pair of conjugate complex roots with positive real parts, indicating that the equilibrium points $E_2$ and $E_3$ are saddle-focus equilibrium points with index 2.

## Effect of variable parameters on the dynamic behavior of the new system

**Figure 2:**
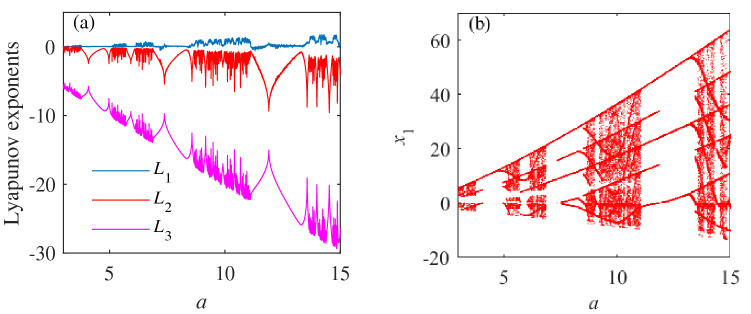
The dynamic behavior when the initial values of system (2) are selected (0.1,0.1,0.1) and $a\in [3,15]$. (**a**) Diagram of numerical changes in the Lyapunov exponent $L_1$,$L_2$, and $L_3$. (**b**) Bifurcation diagram.

We study the dynamic behavior of system (2) under the changes of unknown parameters. The initial value is selected as (0.1,0.1,0.1). Parameter *a* is taken as the control parameter, and its Lyapunov exponent and bifurcation diagram are shown in Fig. 2a,b respectivly. The system’s dynamic characteristics are analyzed in Table 3.

**Table 3:** The range of parameter *a* and the corresponding chaotic state.

The value interval of parameter *a*	Chaos state of system
$a\in [3,3.78]$	Chaotic state
$a\in [3.78,5.12]$	Periodic state
$a\in [5.12,6.12]$	Chaotic state
$a\in [6.12,8.7]$	Period-doubling state
$a\in [8.7,11]$	Chaotic state
$a\in [11,13.6]$	Period-doubling state
$a\in [13.6,15]$	Chaotic state

**Figure 3:**
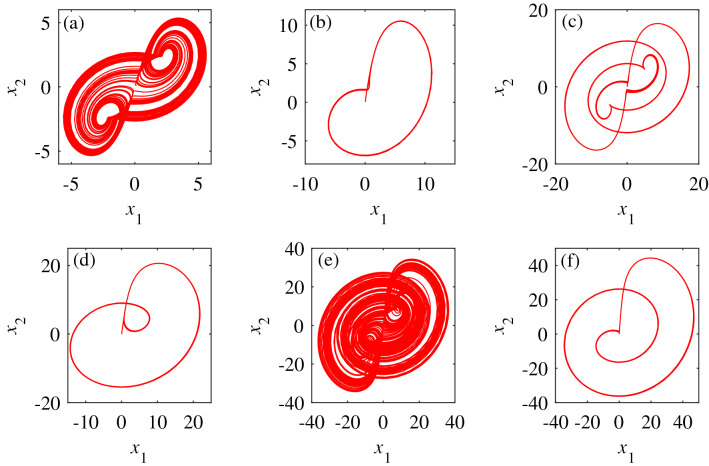
The phase diagrams of system (2) when the initial value is (0.1,0.1,0.1) and the unknown parameters are selected with different values. (**a**) The system phase diagram when *a*=3. (**b**) The system phase diagram when *a*=4.5. (**c**) The system phase diagram when *a*=6. (**d**) The system phase diagram when *a*=10. (**e**) The system phase diagram when *a*=6. (f) Phase diagram of the system at *a*=12.

When $a\in [3,15]$, the numerical changes of the Lyapunov exponents $L_1$, $L_2$, and $L_3$ in Fig. 2a indicate that the dynamic behavior of system (2) is complex, and there are periodic, double-period, and chaotic transitions. Figure 2 and Table 2 show that system (2) is in a chaotic state when $a\in [3,3.78]$, $a\in [5.12,6.12]$, $a\in [8.7,11]$ and $a\in [13.6,15]$; in a periodic state when $a\in [3.78,5.12]$; and in a period-doubling state when $a\in [6.12,8.7]$, and $a\in [11,13.6]$. Figure 3 depicts the attractor diagrams of the special value point of parameter *a*.

The value of parameter *a* increases from 3, and there is an internal crisis bifurcation in $a\cong {3.78}$, $a\cong {8.7}$ and $a\cong {13.6}$. The chaotic attractor collides with the unstable periodic orbit in the attractor basin, causing the attractor to increase. Comparing the Lyapunov exponent diagram, the attractor is in a period-doubling state at the critical point of period and chaos. At $a\cong {7.1}$, the system appears jump bifurcation, and there is a phenomenon of transition from period doubling to period doubling. When the value of parameter *a* begins to decrease from 15, there is a tangent bifurcation phenomenon in $a\cong {11}$ and $a\cong {6.12}$. At this time, the stable nodes and saddle points of the system are combined or separated to produce orbits with chaotic periods and fixed oscillation periods^44^.

By comparing Fig. 3 with Fig. 2, system (2) has the mutual conversion among periodic state, period doubling state and chaotic state when the values of unknown parameters are different, and the trajectories are quite different.

## The effect of the number of unknown parameters on the system dynamic behavior

System (2) contains four unknown parameters with consistent value changes. The change of the value of unknown parameters will cause the dynamic behavior of the chaotic system to change. Many existing articles literatures^45,46^ study the dynamic behavior of chaotic systems, the change of the system’s dynamic behavior is usually explored by varying the parameter value range of the system. Since the unknown parameters of the chaotic system constructed in this paper vary in the same range, this study changes the number of unknown parameters in system (2) to explore the effect of unknown parameters on the dynamic behavior of system (2).

According to Fig. 3a, system (2) has a chaotic attractor when $a = 3$; therefore, the unknown parameter *a* is assigned $a = 3$. Based on the assigned value of *a* in system (2), the chaotic system with an adjustable number of unknown parameters can be obtained. Now a chaotic system with only one unknown parameter, given by system (6), is selected and compared with a chaotic system with two identical unknown parameters, given by system (7). This demonstrates the effect of unknown parameters on the dynamical behavior of chaotic systems. The obtained chaotic system with only one unknown parameter is shown in system (6); a chaotic system with two identical unknown parameters is shown in system (7).

6 $$\begin{aligned}{} & {} \left\{ \begin{array}{lll} \dot{x_1}=-ax_1+x_2x_3\\ \dot{x_2}=3x_1-x_1x_3\\ \dot{x_3}=-3x_3+x_1^2+3\\ \end{array} \right. \end{aligned}$$

7 $$\begin{aligned}{} & {} \left\{ \begin{array}{lll} \dot{x_1}=-ax_1+x_2x_3\\ \dot{x_2}=ax_1-x_1x_3\\ \dot{x_3}=-3x_3+x_1^2+3\\ \end{array} \right. \end{aligned}$$

**Figure 4:**
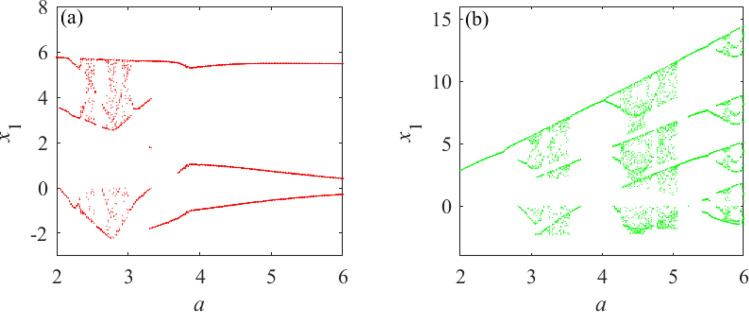
Bifurcation diagrams of systems (6) and (7) under the condition of unknown parameter $a\in [2,6]$ and initial value selection (0.1,0.1,0.1). (**a**) Bifurcation diagram of system (6). (**b**) Bifurcation diagram of system (7).

According to Fig. 2, the value range of the unknown parameter *a* is selected as $a\in [2, 6]$. The bifurcation diagram of Fig. 4 shows the difference between the dynamic behavior of systems (6) and (7), indicating the sensitivity of the chaotic system to the initial conditions.

**Figure 5:**
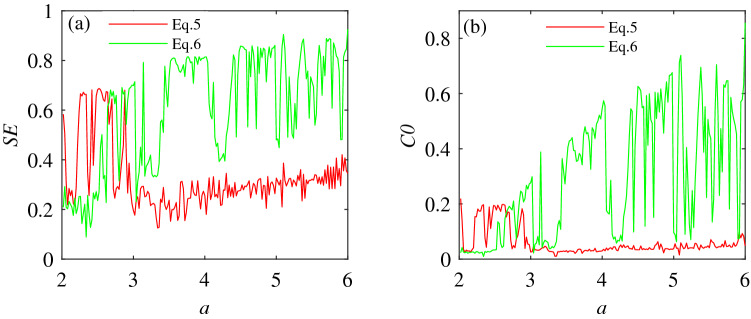
SE complexity and C0 complexity obtained by systems (6) and (7) under the conditions of unknown parameter $a\in [2,6]$ and initial value selection (0.1,0.1,0.1). (**a**) SE complexity. (**b**) C0 complexity.

Figure 4a and b clearly show that the dynamic behavior of the chaotic system (2) has changed significantly after varying the number of the same unknown parameters. To fully illustrate the actual existence of this difference, SE complexity and C0 complexity^47^ are selected to further prove this phenomenon. The complexity measurement provides a certain analysis basis for studying the system’s dynamic behavior. Considering the unknown conditions of parameter $a\in [2,6]$, and initial value (0.1,0.1,0.1), the comparative analysis of systems (6) and (7) complexity is shown in Fig. 5.

From Fig. 4 and Fig. 5, under the premise of determining the value range of unknown parameters for systems (6) and (7), the variation trends of SE complexity and C0 complexity are consistent with the change in the concentration point of the bifurcation diagram. It is further shown that the dynamic behavior of system (2) changes when the number of unknown parameters varies.

## Exponential variation characteristics of nonlinear terms of the new system

### Dynamics change caused by exponential change

By analyzing the dynamic behavior of system (2), the power index of a single variable in the nonlinear term of system (2) is variable. To explore the effect of the power index change of a single variable in the nonlinear term of system (2) on the chaotic system, this paper will illustrate this phenomenon by examples. By adding state variables to system (2), a new system (8) is obtained as follows:

8 $$\begin{aligned} {\left\{ \begin{array}{lll} \dot{x_1}=-ax_1+x_2F_m^i\\ \dot{x_2}=ax_1-x_1F_m^k\\ \dot{x_3}=-ax_3+x_1^2+a\\ \end{array} \right. } \end{aligned}$$

In (8), $F_m^i=x_3^i$, $F_m^k=x_3^k$ and $i,k\in [1,+\infty )$. $F_m^i$ and $F_m^k$ are state variables. The *i* and *k* have the following situations:

9 $$\begin{aligned} {\left\{ \begin{array}{lll} i_{(1)}=k_{(1)}+1,\\ i_{(2)}=k_{(2)}-1,\\ i_{(3)}=k_{(3)}\\ \end{array} \right. } \end{aligned}$$

To explore the effect of $F_m^i$ and $F_m^k$ on the chaotic attractor in system (8), the dissipation degree $\nabla {V}$ of system (8) is calculated:

10 $$\begin{aligned} \nabla {V}=\frac{\partial {\dot{x_1}}}{\partial {x_1}}+\frac{\partial {\dot{x_2}}}{\partial {x_2}}+\frac{\partial {\dot{x_3}}}{\partial {x_3}}=-a+0-a=-2a \end{aligned}$$

The value of *a* is positive, indicating that system (8) will eventually form a dissipation $\nabla {V}$ of the chaotic attractor over time, and that the formation of the chaotic attractor of system (8) is not affected by $F_m^i$ and $F_m^k$.

Now select the appropriate values of *i* and *k* to further explore the dynamic behavior of the chaotic system. Select $i_{(3)}=k_{(3)}=2,3$ respectively to obtain systems (11) and (12):

11 $$\begin{aligned}{} & {} \left\{ \begin{array}{lll} {\dot{x}}_{1}=-ax_1+x_2x_3^2\\ {\dot{x}}_{2}=ax_1-x_1x_3^2\\ {\dot{x}}_{3}=-ax_3+x_1^2+a\\ \end{array} \right. \end{aligned}$$

12 $$\begin{aligned}{} & {} \left\{ \begin{array}{lll} {\dot{x}}_{1}=-ax_1+x_2x_3^3\\ {\dot{x}}_{2}=ax_1-x_1x_3^3\\ {\dot{x}}_{3}=-ax_3+x_1^2+a\\ \end{array} \right. \end{aligned}$$

**Figure 6:**
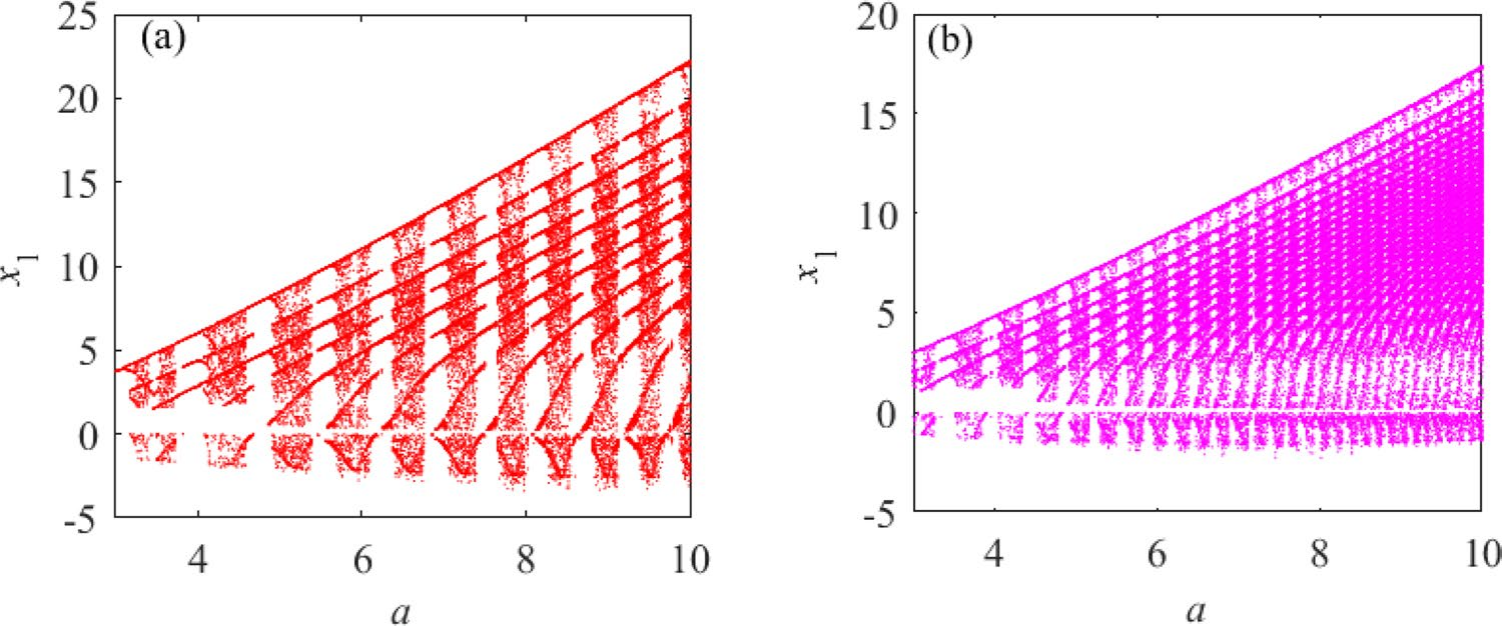
Bifurcation diagrams of systems (11) and (12). (**a**) $i_{(3)}$ = 2, bifurcation diagram of system (11). (**b**) $i_{(3)}$ = 3, bifurcation diagram of system (12).

The initial value is selected as (0.1, 0.1, 0.1), and the range of unknown parameter *a* is $a\in [3,10]$. The bifurcation diagrams of systems (11) and (12) are obtained, as shown in Fig. 6.

**Figure 7:**
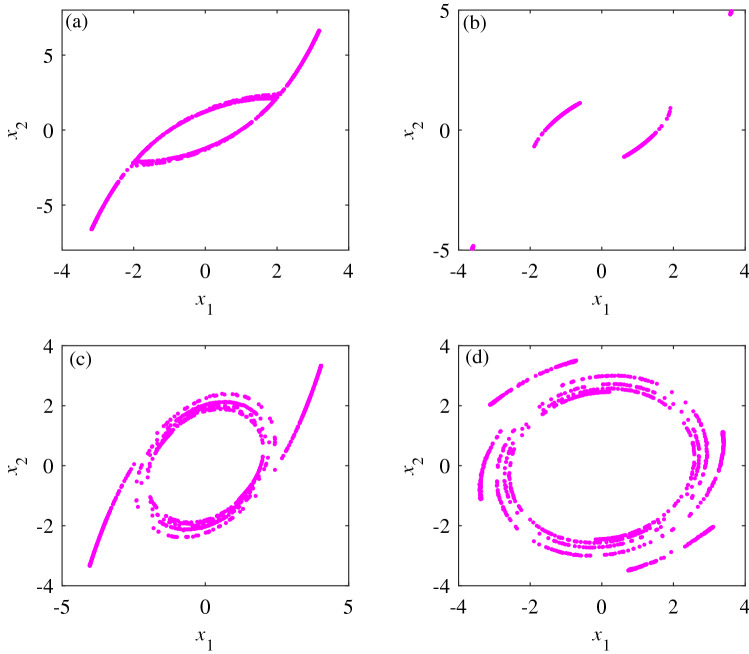
Poincare sections on $x_3=2$ planes. (**a**) poincare section when $i_{(3)}=k_{(3)}=2$ and $a=4.5$. (**b**) poincare section when $i_{(3)}=k_{(3)}=3$ and $a=4.5$. (**c**) poincare section when $i_{(3)}=k_{(3)}=4$ and $a=4.5$. (**d**) poincare section when $i_{(3)}=k_{(3)}=6$ and $a=4.5$.

Figure 6 shows that when $i_{(3)} = k_{(3)} = 2$, the dynamic behavior of system (8) shows the alternation of the period, period doubling and chaos; further, the frequency of alternation varies with the value of *i* and *k*. Simultaneous increases in the value of *i* and *k* result in an increase in the highest power of the system’s nonlinear terms. This changes the chaotic properties of the system as well as the trajectory of the chaotic attractors present in the system. When $i_{(3)} = k_{(3)}$, the value of $i_{(3)}$ and $k_{(3)}$ tend to be infinite, the highest power of the nonlinear terms in system (8) is positive infinity, and the system has chaotic characteristics. To clearly show the dynamic characteristics of system (8) when the value of *i* and *k* change, the Poincare sections of $x_1$ and $x_2$ planes are selected with $a = 4.5$, $i_{(3)}$ = $k_{(3)}$ = 2,3,4, and 6, and $x_3=2$, as shown in Fig. 7.

**Figure 8:**
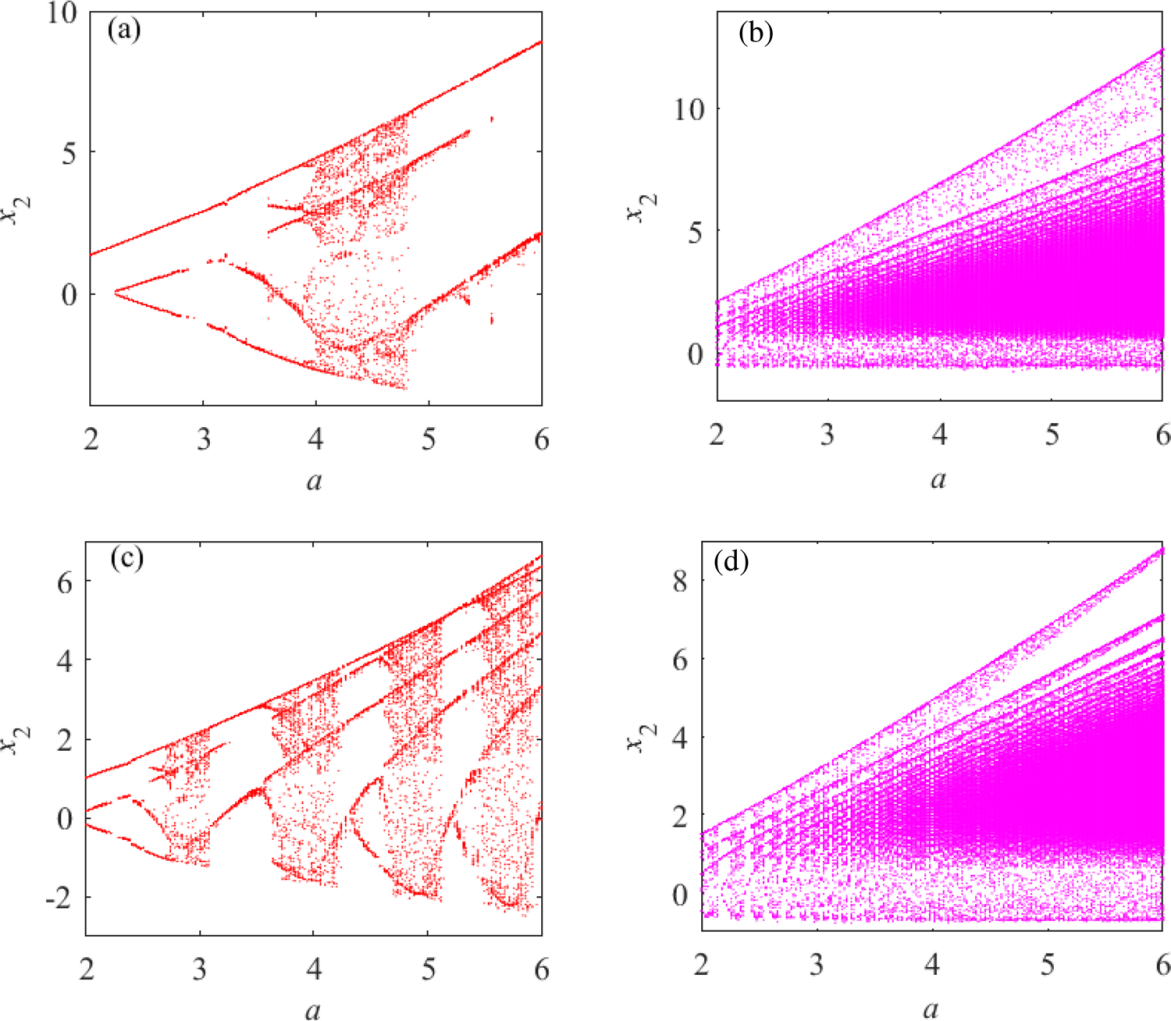
The quantitative relationships between $i_{(m)}$ and $k_{(n)}$ are $i_{(2)}= k_{(2)}-1$ and $i_{(1)}=k_{(1)}+1$ and the bifurcation diagrams of system (8) with different values. (**a**) $i_{(2)}=2$, the bifurcation diagram of system (8). (**b**) $i_{(1)}=2$, the bifurcation diagram of system (8). (**c**) $i_{(2)}=4$, the bifurcation diagram of system (8). (**d**) $i_{(1)}=3$, the bifurcation diagram of system (8).

Select the quantitative relationships between *i* and *k* as $i_{(1)} = k_{(1)} + 1$ and $i_{(2)}$= $k_{(2)}-1$. The initial value of system (8) is (0.1, 0.1, 0.1), the unknown parameter value ranges are $a \in { [2, 6]}$. When $i_{(1)}$= $i_{(2)}$= 2 and $i_{(1)}$ = $i_{(2)}$ = 4 are selected respectively, the bifurcation diagrams of system (8) under two different values are shown in Fig. 8.

Figure 8 shows that in two different states of $i= k$, the chaotic characteristics of system (8) are very different by comparing the presence of dense points in the bifurcation diagram at different times. When the quantitative relationship between *i* and *k* is $i_{(3)}\ne {k_{(3)}}$, the speed of dynamic behavior transformation of $i>{k}$ is faster than that of $i<k$ in the same time. According to Figs. 7 and 8, as the values of *i* and *k* increase, the motion trajectory of the chaotic attractor of system (8) changes; this shows that under certain value conditions, the change of *i* and *k* values will affect the dynamic behavior of the system.

### 0–1 test

To further show the dynamic behavior of the system with different $i_{(m)}$ and $k_{(n)}$ values, this paper uses the 0–1 test method to explore the new system. Here, by comparing the dynamic behavior of the system when the same unknown parameters are taken in systems (11) and (12), we further illustrate the effect of the exponential value change on the dynamic behavior of the system.

The improved 0–1 test algorithm^48,49^ used in this paper defines the following equation:

13 $$\begin{aligned} \left\{ \begin{array}{lll} p(n)=\sum \limits ^{n}_{j=1}\phi (j){\textrm{cos}}(\theta (j)),n=1,2,3\cdots \\ s(n)=\sum \limits ^{n}_{j=1}\phi (j){\textrm{sin}}(\theta (j)),n=1,2,3\cdots \\ \end{array} \right. \end{aligned}$$

In (13), $\phi (j)$ represents an observable dataset and $\theta (j)=jc+\sum \limits _{i=1}^{j}\phi (i)$, $j=1,2,3,\cdots ,n$. On the basis of *p*(*n*), the root mean square displacement is defined:

14 $$\begin{aligned} M(n)=\mathop {\textrm{lim}}\limits _{N\rightarrow \infty }\frac{1}{N}\sum \limits ^{n}_{j=1}[p(j+n)-p(j)]^2,n=1,2,3,\cdots \end{aligned}$$

**Figure 9:**
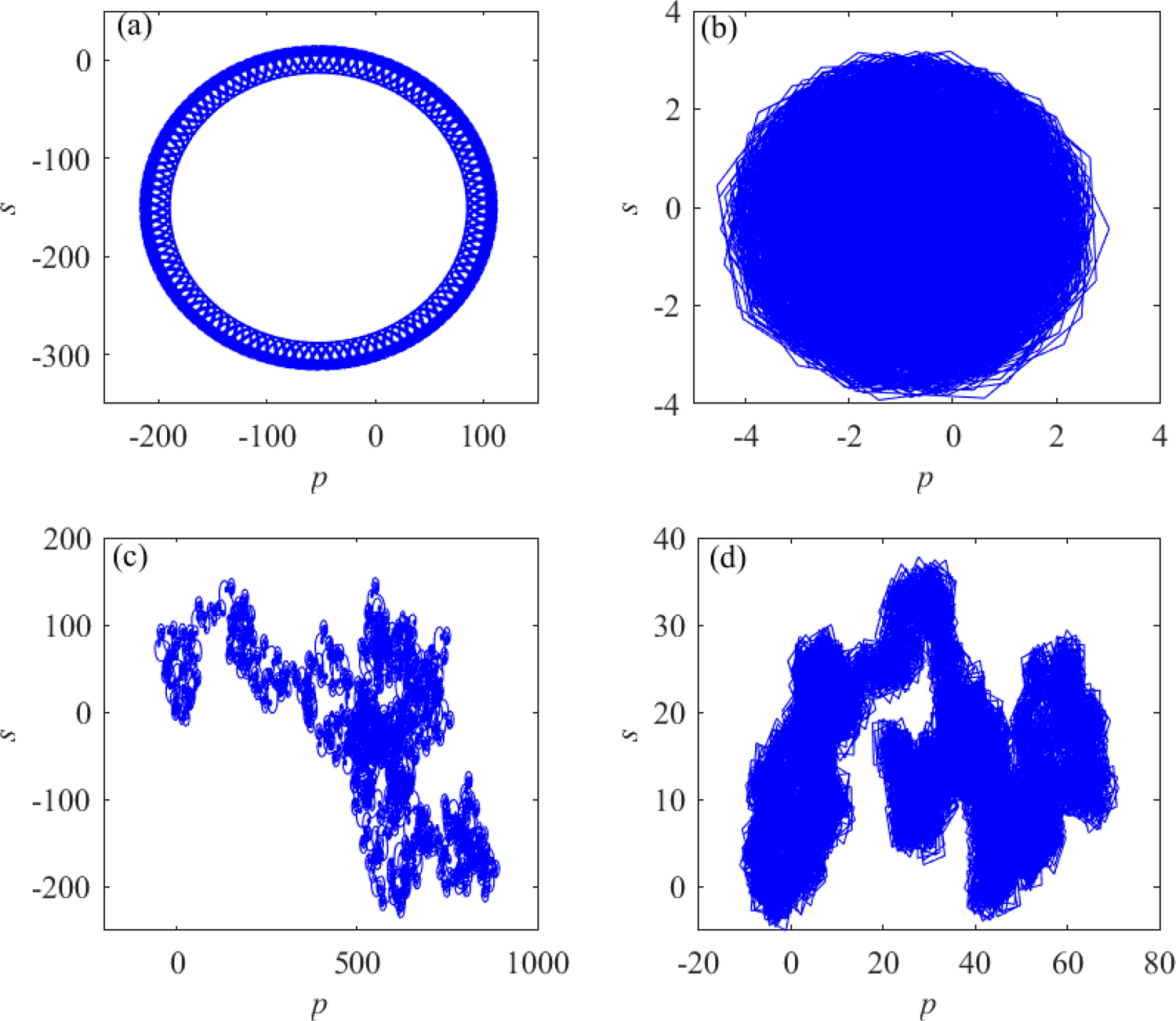
State space diagrams of systems (11) and (12). (**a**) The state space diagram of system (11) when *a*=3. (**b**) The state space diagram of system (12) when *a*=3. (**c**) The state space diagram of system (11) when *a*=6.25. (**d**) State space diagram of system (12) at *a*=6.25.

When the behavior of *p*(*n*) or *s*(*n*) is in Brownian motion, the RMS displacement *m*(*n*) increases linearly with time. When the behavior of *p*(*n*) or *s*(*n*) is bounded, then the RMS displacement *M*(*n*) is also bounded. For systems (13) and (14), 0–1 test is conducted when the values of unknown parameter *a* are set as $a=3$ and $a=6.25$. The resulting state space diagrams are shown in Fig. 9.

Figure 9 depicts the apparent difference between the state space diagram of systems (13) and (14), which corresponds to the dynamic behavior in the bifurcation diagram of Fig. 6. It further indicates that the numerical value of the exponential of the nonlinear term in system (10) change the system’s dynamic behavior.

## Circuit simulation analysis

**Figure 10:**
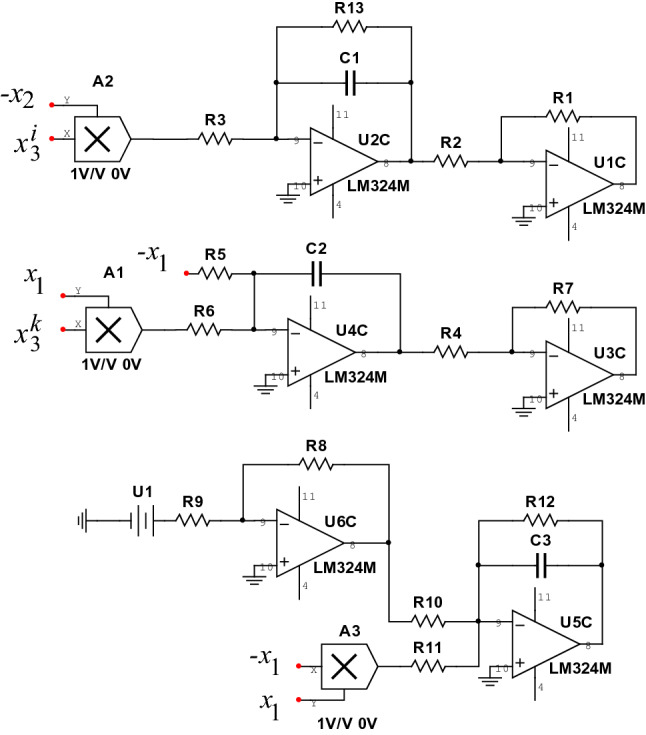
System (8) circuit simulation diagram.

To verify the feasibility of circuit implementation of system (8), the circuit diagram of system (8) is set up when the value range of index $i_{(m)}$ and $k_{(n)}$ are positive natural number as shown in Fig. 10.

The operational amplifier (LM324M) and other related components are used for related operations such as addition, subtraction and integration. Apply Kirchhoff’s law to Fig. 10 to get the differential equation:

15 $$\begin{aligned} {\left\{ \begin{array}{lll} \dot{x}_1=-\frac{1}{C_1R_{13}}x_1+\frac{R_7}{C_1R_3R_4}x_2x_3^{i}\\ \\ \dot{x}_2=\frac{R_1}{C_2R_2R_5}x_1-\frac{1}{C_2R_6}x_1x_3^{k}\\ \\ \dot{x}_3=-\frac{1}{C_3R_{12}}x_3+\frac{R_8}{C_3R_9R_{10}}{U_1}+\frac{R_1}{C_3R_2R_{11}}x_1^2\\ \end{array} \right. } \end{aligned}$$

The capacitors are set to $C_1=C_2=C_3=1\textrm{uF}$, and the other corresponding resistance values of system (15) are as follows:

16 $$\begin{aligned} \left\{ \begin{array}{lll} R_3=R_6=R_{11}=1000{\textrm{k}{\Omega }}\\ R_1=R_2=R_4=R_7=10{\textrm{k}{\Omega }}\\ R_8=1{\textrm{k}{\Omega }},R_9=10{\textrm{k}{\Omega }},R_{10}=100{\textrm{k}{\Omega }}\\ \end{array} \right. \end{aligned}$$

**Figure 11:**
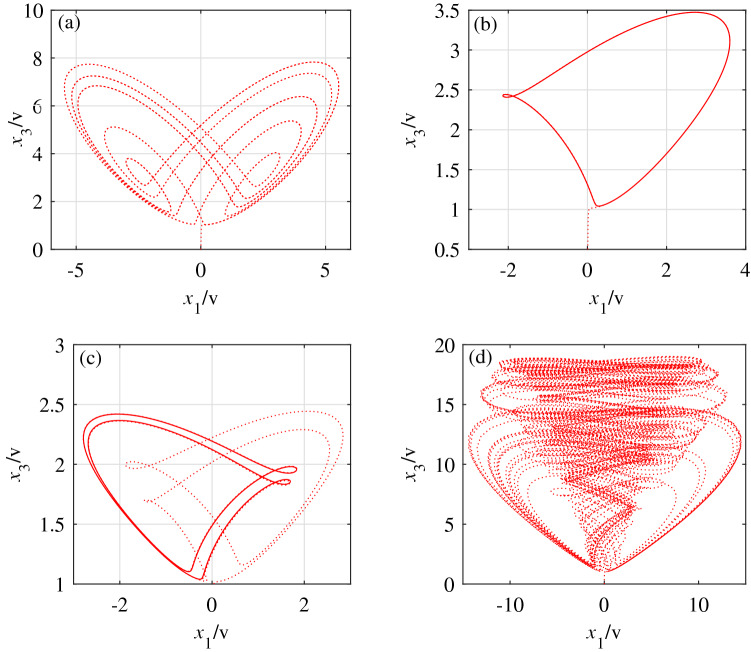
Attractor diagrams of $x_1-x_3$ plane at various times. (**a**) When $i_{(3)}=k_{(3)}=1$, the circuit simulation diagram of the $x_1-x_3$ plane. (**b**) When $i_{(3)}=k_{(3)}=2$, the circuit simulation diagram of the $x_1-x_3$ plane. (**c**) When $i_{(3)}=k_{(3)}=3$, the circuit simulation diagram of the $x_1-x_3$ plane. (**d**) When $i_{(1)}=k_{(1)}+1=2$, the circuit simulation diagram of the $x_1-x_3$ plane.

Among them, the values of $R_5$, $R_{12}$, $R_{13}$ and the voltage $U_1$ can be adjusted to match the value of the unknown parameter *a* in system (8). When the unknown parameter is $a=3$, select $R_5= R_{12}= R_{13}=333.33\textrm{k}{\Omega }$, $U_1=3\textrm{V}$, a multiplier (AD633) with an output gain of 1, and the exponent of the state variable $x_3$ of the nonlinear term in system (8) as a variable parameter. The circuit simulation diagrams of parameters *i* and *k* with different values are obtained, as shown in Fig. 11.

Figure 11 shows that the simulation effect matches the dynamic behavior of the corresponding period. The dynamic behavior of system (8) under different initial conditions is further verified, which provides a theoretical reference for the hardware implementation of system (8).

## Image encryption processing

To explore the relative performance of encryption and decryption exhibited by the chaotic system with the same unknown parameters applied to the image encryption system, this paper applies system (2) to the chaotic image encryption system with the same encryption and decryption process.

**Figure 12:**
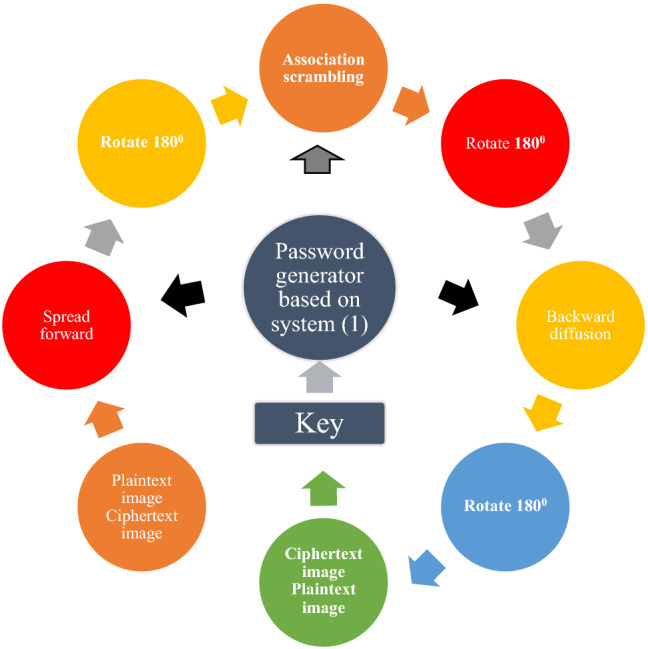
Image encryption system with the same encryption process and decryption process based on system (2).

The image encryption system with the same encryption process and decryption process adopted in this paper mainly includes five parts: cipher generation, forward diffusion, correlation scrambling, image rotation and backward diffusion. Among them, the encryption system uses a scrambling algorithm associated with plaintext. A schematic diagram of the structure of the image cryptography system based on system (2) is shown in Fig. 12.

Given that the plaintext image *P* size is $M\times {N}$, the gray level is *L* bits, and $\textrm{mod} (N,2)=0$ is satisfied. When $\textrm{mod} (N,2)\ne 0$, it is necessary to add a column vector of all 0s to the $M\times {1}$ column of image *P* to obtain a new image of size $M\times (N+1)$. The selected key is $K=[x_0,y_0,z_0,r_1,r_2]$, where $x_0,y_0,z_0$ are the initial value of system (2), and $r_1$ and $r_2$ are the 8-bit random number. The password generation module application generates 6 pseudo-random matrices, denoted as *X*, *Y*, *Z*, *V*, *U*, and *W* with size of $M\times {N}$.

- **Step 1**: Select $x_0,y_0,z_0$ in the key *K* as the initial value of system (2), and iterate system (2) $r_1+r_2+MN$ times to obtain 3 pseudo-random sequences $x_i$, $y_i$, $z_i$, $i=1,2,3\cdots {MN}$
- **Step 2**: Apply the following equation to the pseudo-random sequence. ${x_i}$, $y_i$, ${z_i}$ and generate the matrix *X*, *Y*, *Z*, *G*, *U*,  and *Q*.
  17 $$\begin{aligned} \left\{ \begin{array}{lll} X(u,v)={\mathrm{mod(floor}}(x_{(u-1)\times {N}}\times {10^{15}}),2^L)\\ Y(u,v)={\mathrm{mod(floor}}(y_{(u-1)\times {N}}\times {10^{14}}),2^L)\\ Z(u,v)={\mathrm{mod(floor}}(z_{(u-1)\times {N}}\times {10^{13}}),N)+1\\ G(u,v)={\mathrm{mod(floor}}(x_{(u-1)\times {N}}+z_{(u-1)\times {N}}\times {10^{12}}),M)+1\\ U(u,v)={\mathrm{mod(floor}}(y_{(u-1)\times {N}}+z_{(u-1)\times {N}}\times {10^{11}}),M)+1\\ Q(u,v)={\mathrm{mod(floor}}(y_{(u-1)\times {N}}+y_{(u-1)\times {N}}\times {10^{10}}),M)+1\\ \end{array} \right. \end{aligned}$$
- **Step 3**: Use the pseudo-random matrix *X* and $r_1$ to perform forward diffusion processing on the plaintext image *P*, and apply the exclusive OR operation to obtain the matrix *A*:
  18 $$\begin{aligned} \left\{ \begin{array}{lll} A(1,1)=P(1,1)\oplus {X(1,1)}\oplus {r_1}\\ A(1,j)=P(i,1)\oplus {X(i,1)}\oplus {E(1,j-1)}\\ A(i,1)=P(i,1)\oplus {X(i,1)}\oplus {E(i-1,N)}\oplus {E(i-1,1)}\\ A(i,j)=P(i,j)\oplus {X(i,j)}\oplus {E(i-1,j)}\oplus {E(i,j-1)}\\ \end{array} \right. \end{aligned}$$
  In (18), $$i=2,\cdots ,$$ $$M,j=2,\cdots ,N$$.
- **Step 4**: Rotate the matrix *A* $180^0$ to obtain the matrix $\theta$.
- **Step 5**: Using pseudo-random matrices *Z*, *G*, *U* and *Q* to scramble the explicit association of matrix $\theta$, the method is as follows:
  Replace the pixel position $\theta =(i, j)$ with $\theta =({\overline{k}},{\overline{s}})$. If mod(*N*,2)=0, then ${\overline{k}}$ and ${\overline{s}}$ are calculated according to system (19).
  19 $$\begin{aligned} \left\{ \begin{array}{lll} \bar{k}=(M+1)-({\textrm{mod}}(U(i,j))+{\textrm{sum}}(\theta (G(i,j),1:N)),M)+1)\\ \bar{s}=(N+1)-({\textrm{mod}}(Q(i,j))+{\textrm{sum}}(\theta (1:M,Z(i,j))),N)+1)\\ \end{array} \right. \end{aligned}$$
  If mod$(N,2)\ne {0}$, then use the following system (20) to calculate $\bar{k},\bar{s}$.
  20 $$\begin{aligned} \left\{ \begin{array}{lll} \bar{k}={\textrm{mod}}(U(i,j)+{\textrm{sum}}(\theta {(G(i,j),1:N)}),M)+1\\ \bar{s}={\textrm{mod}}(Q(i,j)+{\textrm{sum}}(\theta {(1:M,Z(i,j))}),N)+1\\ \end{array} \right. \end{aligned}$$
  **Step 6**: When $\bar{k}=i$, or $\bar{s}=j$, or ${\bar{k}}= V(i,j)$, or $\bar{s}=Z(i,j)$, or $G(i,j)=i$, or $Z(i,j)=j$, keep the position of $\theta (i,j)$ unchanged, otherwise replace $\theta =(i, j)$ with $\theta =(\bar{k},\bar{s})$.
- **Step 7**: According to the order from left to right and top to bottom, process the position of each pixel of matrix $\theta$, and repeat **Step 5** and **Step 6**.
  The scrambling algorithm used in the encryption and decryption processes is the same, but the cipher matrix is different. The cipher matrix corresponding to the decryption process is as follows:
  21 $$\begin{aligned} \left\{ \begin{array}{lll} &{}\widetilde{X}={\textrm{rot}}180(X),\widetilde{Y}={\textrm{rot}}180(Y),\widetilde{Z}=(N+1)-{\textrm{rot}}180(Z)\\ &{}\widetilde{Q}={\textrm{rot}}180(W),\widetilde{U}={\textrm{rot}}180(U),\widetilde{G}=(M+1)-{\textrm{rot}}180(V)\\ \end{array} \right. \end{aligned}$$
- **Step 8**: Perform $180^0$ rotation processing on the scrambled matrix $\theta$ to obtain the matrix *F*.
- **Step 9**: Use pseudo-random matrices *Y* and $r_2$ to perform backward diffusion processing on the plaintext image *F*, and apply the exclusive OR operation to obtain the matrix *H*:
  22 $$\begin{aligned} \left\{ \begin{array}{lll} H(M,N)=F(M,N)\oplus {Y(M,N)}\oplus {r_2}\\ H(M,j)=F(M,j)\oplus {Y(M,j)}\oplus {F(M,j+1)}\\ H(i,N)=F(i,N)\oplus {Y(i,N)}\oplus {F(i+1,1)\oplus {F(i+1,N)}}\\ H(i,j)=F(i,j)\oplus {Y(i,j)}\oplus {F(i+1,j)\oplus {F(i,j+1)}}\\ \end{array} \right. \end{aligned}$$
  In (22), $$i=M-1,\cdots ,1,$$ $$j=N-1,\cdots ,1$$.
- **Step 10**: Rotate the matrix *H* $180^0$ to obtain the matrix *B*, which is the ciphertext image..

**Figure 13:**
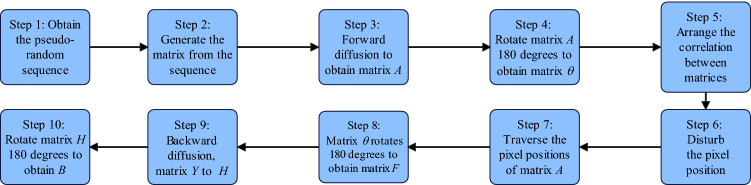
Encryption process diagram.

The encryption process is summarized in Fig. 13. The encryption process of the algorithm is the same as the decryption process. At the same time, unlike the classical image cryptosystem, the algorithm does not have a loop operation, only contains two diffusion and a scrambling operation. Since only the scrambling algorithm associated with the plaintext is used, the diffusion algorithm has nothing to do with the plaintext. Thus the confidentiality effect is stronger.

## Simulation results and performance analysis

### Encryption and decryption of images

This section mainly analyzes the relevant encryption and decryption performance of the grayscale diagram of this chaotic encryption system. All of the following simulation experiments are implemented on Matlab2017b and the computer’s associated configuration of 12GB RAM and Intel(R)Core(TM) i5-7200U CPU @ 2.50GHz 2. The simulation used a grayscale map of Brick Wall, Sand, Motion, Grass, and Toy Vehicle, with all five images having 512 $\times$ 512 pixels.

**Figure 14:**
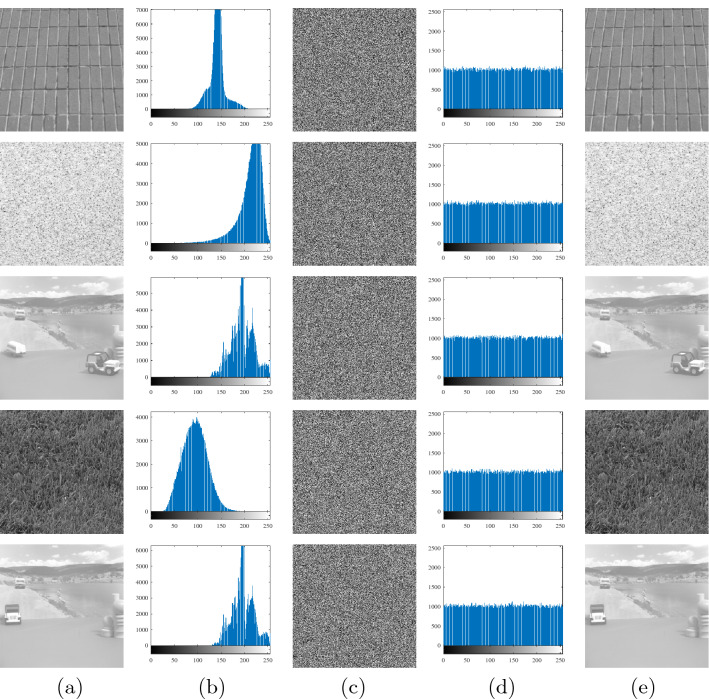
Plaintext images, encrypted images, decrypted images, and histograms of Brick Wall, Sand, Motion, Grass, and Toy Vehicle. (**a**) Plaintext images. (**b**) Histogram of the plaintext images. (**c**) Encrypted images. (**d**) Histogram of the encrypted images. (**e**) Decrypted images.

The key $K=[x_0,y_0,z_0,r_1,r_2]$ is selected, where $x_0,y_0,$ and $z_0$ are the initial values of system (2), and $r_1$ and $r_2$ are the 8-bit random number ($r_1,r_2\in [0,255]$). The unknown parameter *a* of system (2) is assigned of $a=3$. The key is selected as $x_0,y_0,z_0\in [-50,50]$, and the step length is selected as 1/*t*, where $t=10^{14}$. The size of the key space is 6.5536$\times {10^{52}}$. The key is selected as $K=[ -40.1, 40.1, -35.7, 100.0, 235.0]$ to obtain plaintext, encrypted and decrypted images based on system (2), as shown in Fig. 14.

Figure 14 shows that the encryption and decryption effect of the encryption system is remarkable. In the figure, the histogram of the ciphertext rendering is evenly distributed, which can effectively resist the attack and obtain useful information.

### Encryption and decryption time test

The image encryption system is ultimately applied to actual life, and there are specific requirements for the encryption and decryption time. The encryption and decryption time of the image encryption system must be efficient and fast. The article tests the encryption and decryption time of different pixel grayscale maps twenty times and chooses the average. The resulting test results are shown in Table 4.

**Table 4:** Time test of encryption and decryption (in seconds).

Image size	$128\times {128}$	$256\times {256}$	$512\times {512}$	$1024\times {1024}$
Encryption time	0.165126	0.699598	2.491453	10.363338
Decryption time	0.177105	0.599330	2.864108	10.400929

The time of encryption and decryption of the grayscale map under different pixels shown in Table 4 can better show the superiority of the encryption system in the encryption and decryption time and has a great possibility of being applied to the production practice.

### NIST test

Chaotic pseudo-random sequences are an important part of the chaotic image cryptography system, and the password of the image encryption system has excellent statistical characteristics. Typically, chaotic sequences used for image encryption must pass the pseudo-random sequence test. NIST^50,51^ is a typical test method, and the test results are authoritative. The chaotic sequence used for the image encryption system has strong statistical characteristics; this is the basis for the perfect success of the image encryption system, NIST test for the chaotic sequences is selected for this article. Table 5 shows the test results.

**Table 5:** NIST test results for chaotic systems.

No.	Test name	*P*-value	Results
1	Frequency	0.7991	Success
2	Block frequency	0.2272	Success
3	Runs	0.8434	Success
4	Longest run	0.2138	Success
5	Rank	0.1575	Success
6	FFT	0.6464	Success
7	Non-overlapping template	0.8884	Success
8	Overlapping template	0.4653	Success
9	Universal	0.2326	Success
10	Linear complexity	0.5170	Success
11	Serial	0.2185	Success
12	Approximate entropy	0.9333	Success
13	Cumulative sums	0.9867	Success
14	Random excursions	0.3803	Success
15	Random excursions variant	0.2673	Success

Table 5 shows that the chaotic sequence successfully passed 15 test experiments, which proves that the chaotic sequence used in the image encryption system in this paper has excellent statistical characteristics.

### $\chi ^2$ test

Figure 14 shows that there are obvious differences between the histogram of the plaintext image and the histogram of the ciphertext image. The histogram image of the plaintext image is irregular, and the histogram of the encrypted image is relatively flat. To further explore the quantitative difference between the histogram of a plaintext image and the histogram of a redaction image, the $\chi ^2$ statistic (unilateral hypothesis detection) is used to measure the quantitative difference between them. Using the Pearson $\chi ^2$ statistic Eq. (23) follows a $\chi ^2$ distribution with $n-1$ degrees of freedom. Image size is $M\times {N}$, assuming that grayscale pixels $f_i$ in the histogram follow an even distribution with $i=0,1,2,\cdots ,255$.

23 $$\begin{aligned} \chi ^2=\sum ^{255}_{i=0}\frac{(f_i-g)^2}{g},\ \ g=(M\times {N})/256 \end{aligned}$$

The significance levels $\alpha =0.05$ and $\chi ^2_{0.05}=284.33591$ are selected. The $\chi ^2$ test results for plaintext images and ciphertext images of Brick Wall, Sand, Motion, Grass, and Toy Vehicle are shown in Table 6.

**Table 6:** The $\chi ^2$ test results.

Image	Brick Wall	Sand	Motion	Grass	Toy vehicle
Plain image	$1.7234\times 10^6$	$7.4641\times 10^5$	$1.1495\times 10^6$	$4.5668\times 10^5$	$1.3194\times 10^6$
Cipher image	271.9492	244.9160	267.2324	264.7871	258.2129
Pass or Fail	Pass	Pass	Pass	Pass	Pass

The calculated values of the $\chi ^2$ statistic of the five plaintext images in Table 6 are significantly greater than that of $\chi ^2_{0.05}(255)$, and the calculated values of the $\chi ^2$ statistic of the ciphertext images are significantly less than that of $\chi ^2_{0.05}(255)$; this can be considered to be approximately evenly distributed in the histogram of the ciphertext image in Fig. 14, indicating that the encryption algorithm can resist the attack well.

### Information entropy analysis

Information entropy reflects the uncertainty of image information to a certain extent. In general, the larger the value of information entropy, the less visual information. The equation for calculating the expression of information entropy is:

24 $$\begin{aligned} H=-\sum ^L_{i=0}p(i){\textrm{log}}_2p(i) \end{aligned}$$

where *L* is the number of gray levels of the image and *p*(*i*) is the probability of gray value *i* appearing.

The images are selected as 8 bits, and the outstanding value of information entropy is 8. The entropy of information in plaintext and ciphertext is calculated, and the table of changes in information entropy is shown in the Table 7.

**Table 7:** Information entropy test.

Image	Plain image	Cipher image	Theoretical value
Brick wall	5.6826	7.9993	8.0000
Sand	6.4154	7.9993	8.0000
Motion	6.2635	7.9993	8.0000
Grass	6.7359	7.9993	8.0000
Toy Vehicle	6.1422	7.9993	8.0000

Table 7 shows the values of the entropy of plaintext images and ciphertext images. The information entropy value of the plaintext is quite different from the theoretical value, and the information entropy value of the ciphertext is close to the ideal information entropy value, indicating that the encryption system has a better encryption effect on system (2).

### Key sensitivity analysis

Key sensitivity is mainly used to analyze the difference between two ciphertext images obtained by encrypting the same plaintext image when the key changes in the image encryption system. Since a good image encryption system has strong key sensitivity, it is particularly important to test the key sensitivity of the image encryption system.

In this section, we select the initial value of system (2) as $K = [x_0, y_0, z_0, r_1, r_2]$, where $x_0, y_0, z_0\in [50, 50]$, $r_1$ and $r_2 \in [0, 255]$, at steps of $10^{-14}$. As a test for *K*, 1000 values are randomly selected from the keyspace. For each set of keys to vary a specific number of the variable, each change is in a step of $10^{-14}$. Further, the same plaintext image is encrypted with the key before and after the change in order to compare the two ciphertext images obtained. NPCR and UACI^52^ must investigate the performance indicators for image encryption, which is defined as: assuming that two plaintext images $P_1$ and $P_2$ are the same except for the value difference of 1 at a pixel (*q*, *p*), the same chaotic encryption system is used to encrypt the plaintext image to obtain the corresponding ciphertext images $C_1$ and $C_2$:

25 $$\begin{aligned}{} & {} G(q,p) = \left\{ \begin{array}{lll} 0,\quad C_1(q,p)=C_2(q,p)\\ 1,\quad C_1(q,p)\ne C_2(q,p)\\ \end{array} \right. \end{aligned}$$

26 $$\begin{aligned}{} & {} \left\{ \begin{array}{lll} {\textrm{NPCR}}=\frac{1}{M\times {N}}\sum \limits ^M_{q=1}\sum \limits ^N_{p=1}{G(q,p)}\times 100\%\\ {\textrm{UACI}}=\frac{1}{M\times {N}}\sum \limits ^M_{q=1}\sum \limits ^N_{p=1}\frac{\vert C_1(q,p)-C_2(q,p)\vert }{255-0}\times 100\%\\ \end{array} \right. \end{aligned}$$

According to the calculated value given in Ref.^53^, the theoretical value of NPCR is 99.5893% when the plaintext image is $512\times 512$, and the theoretical value interval of UACI is (33.3730%, 33.5541%). Use the Brick Wall, Sand, Motion, Grass and Toy Vehicle image to test. The calculated values for NPCR and UACI are shown in Table 8.

**Table 8:** Key sensitivity analysis.

Variable	Index	Brick Wall	Sand	Motion	Grass	Toy Vehicle	Theoretical value
$x_0$	NPCR	99.6189	99.6231	99.6063	99.6201	99.6056	99.5893
	UACI	33.4018	33.4842	33.4541	33.4112	33.4863	(33.3730, 33.5541)
$y_0$	NPCR	99.6231	99.6105	99.6193	99.6155	99.6140	99.5893
	UACI	33.4346	33.5065	33.5194	33.4564	33.3789	(33.3730, 33.5541)
$z_0$	NPCR	99.6292	99.6025	99.6048	99.6037	99.6223	99.5893
	UACI	33.3678	33.4720	33.5033	33.3687	33.4449	(33.3730, 33.5541)
$r_1$	NPCR	99.6109	99.5956	99.6071	99.5796	99.5972	99.5893
	UACI	33.3371	33.4980	33.4579	33.3419	33.4560	(33.3730, 33.5541)
$r_2$	NPCR	99.6117	99.6105	99.6101	99.5995	99.5834	99.5893
	UACI	33.4946	33.4464	33.3868	33.5322	33.4564	(33.3730, 33.5541)

**Figure 15:**
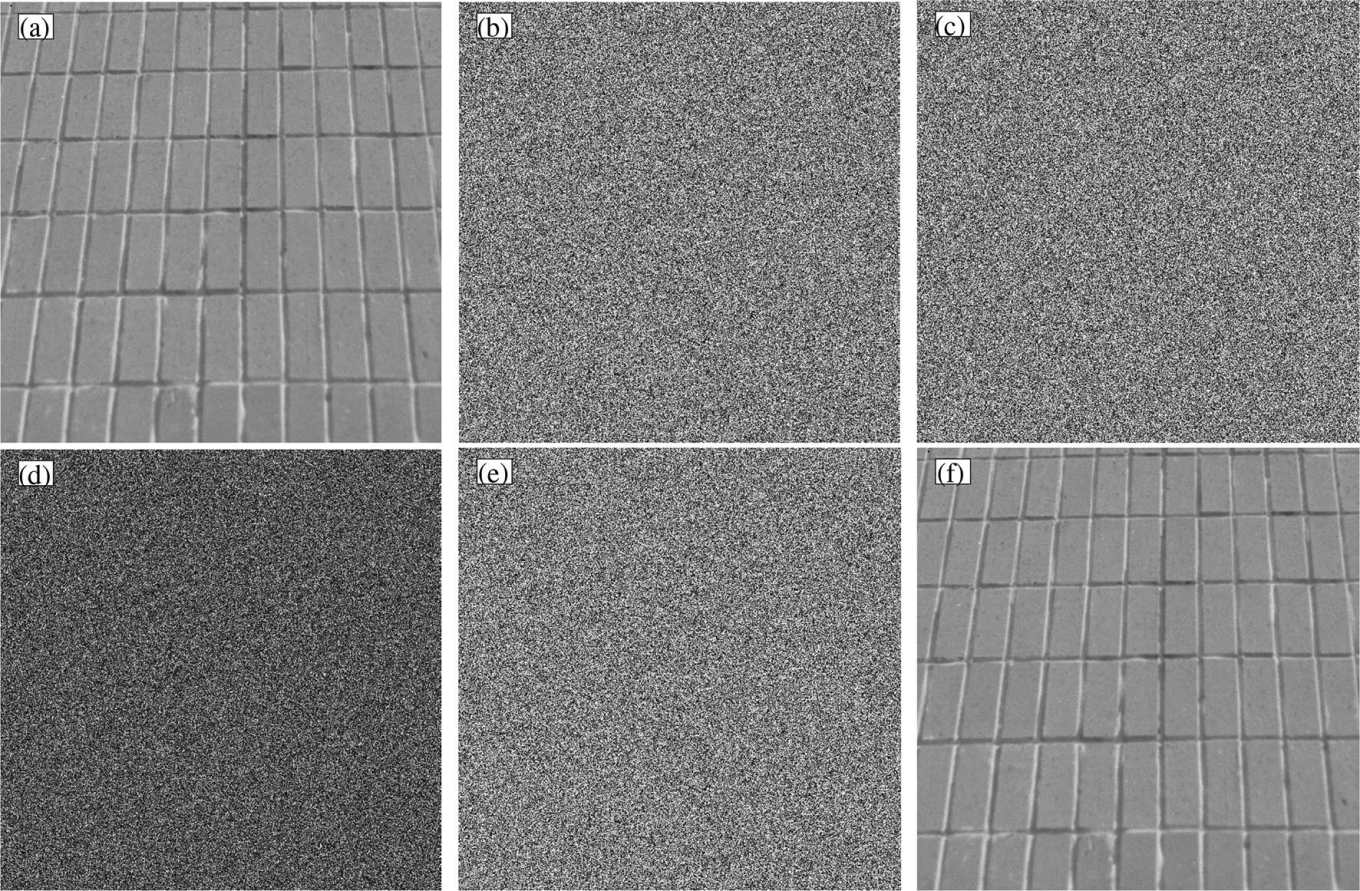
Key sensitivity test results. (**a**) Plaintext. (**b**) Ciphertext under $K_a$. (**c**) Cipher text under $K_b$. (**d**) The ciphertext difference between $K_a$ and $K_b$. (**e**) Decryption under $K_b$. (**f**) Decryption under $K_a$.

The test results of NPCR and UACI in Table 8 all meet the theoretical numerical requirements, indicating that all passed the test. To further explore the sensitivity of the key in the encryption and decryption process, we select a set of $K_a=[-35.5,-15.4,25.6,111,222]$ in the key space, and add an increment to it to obtain $K_b=[-35.5+10^{-14},-15.4,25.6,111,222]$ as the wrong key. Use the Brick Wall image to test, and the test results are shown in Fig. 15.

Through the key sensitivity test, Fig. 15 shows that when there is a small error in the value of the key space, the decryption result will be biased, resulting in a mismatch between the decrypted image and the plaintext image. The key space is strongly sensitive to the value of the key.

### Noise attack detection

**Figure 16:**
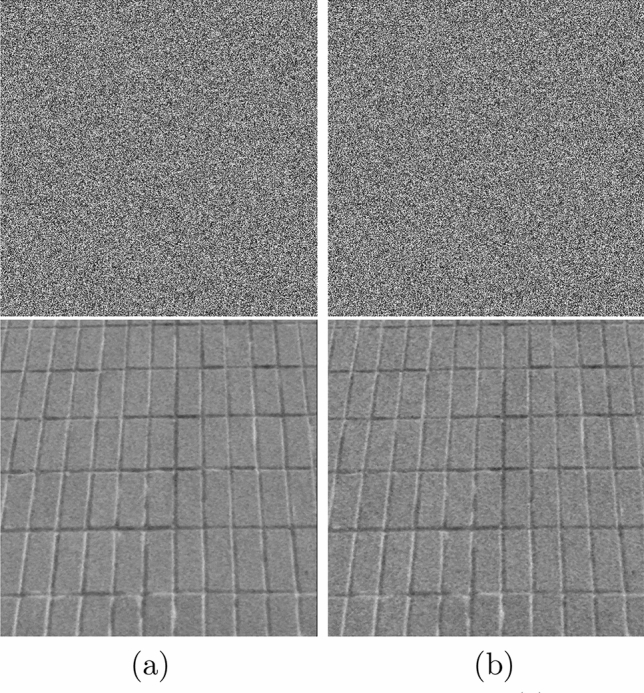
Encrypted and decrypted images for noise attack. (**a**) Salt and pepper noise of intensity 0.05. (**b**) Salt and pepper noise of intensity 0.1.

To further explore the anti-interference performance of the chaotic encryption system proposed in this paper, the noise intensity of 0.05 and 0.1 is added to the encryption and decryption test, and the obtained anti-interference effect diagram is shown in Fig. 16.

Figure 16 shows the decrypted image obtained by decoding the encrypted image with different intensities of salt and pepper noise has excellent discrimination. The encryption algorithm is robust to noise attacks and has excellent security performance.

### Clear text sensitivity analysis

With the help of the same key, the plaintext sensitivity test uses an image encryption system to encrypt two slightly different plaintext images. The corresponding ciphertext image is obtained, and the sensitivity of the image encryption system to the plaintext is reflected by comparing the differences in the obtained ciphertext images.

To test the sensitivity of the image encryption system used in this article to clear text, the classic plaintext image is selected as the experimental test image. Select $K_a=[-35.5,-15.4,25.6,111,222]$, which compares the values of NPCR and UACI of two plaintext images by varying the value of a pixel in the plaintext image and encrypting it again. Through repeated calculations, the plaintext sensitivity analysis results of the chaotic encryption system are shown in Table 9.

**Table 9:** Clear text sensitivity analysis.

Index	Brick wall	Sand	Motion	Grass	Toy vehicle	Theoretical value
NPCR	99.6006	99.5937	99.6174	99.6052	99.5972	99.5893
UACI	33.5265	33.4227	33.4926	33.4093	33.3974	(33.3730, 33.5541)

After analyzing the clear text sensitivity results of the chaotic encryption system in Table 9, the calculation results of NPCR and UACI are similar to the theoretical values after the plaintext images with minor pixel differences are encrypted. The results illustrates that the chaotic image encryption system proposed in this paper has prominent plaintext sensitivity.

### Correlation analysis of encryption system

To show the correlation between adjacent pixels after the encryption system processes the plaintext, we randomly select $\beta$ pairs of adjacent pixels from the used image. The gray value is $(\mu _i,\lambda _i)$, $i=1,2,3,\cdots ,\beta$, and then obtain the correlation coefficient *T*.

27 $$\begin{aligned}{} & {} T=\frac{{\textrm{cov}}(\mu ,\lambda )}{\sqrt{D(\mu )}\sqrt{D(\lambda )}} \end{aligned}$$

28 $$\begin{aligned}{} & {} {\textrm{cov}}(\mu ,\lambda )=\beta ^{-1}\sum ^{\beta }_{i=1}(\mu _i-E(\mu ))(\lambda _i-E(\lambda )) \end{aligned}$$

29 $$\begin{aligned}{} & {} D(\rho )=\beta ^{-1}\sum ^\beta _{i=1}(\rho _i-E(\rho ))^2 \end{aligned}$$

30 $$\begin{aligned}{} & {} E(\rho )=\beta ^{-1}\sum ^\beta _{i=1}\rho _i \end{aligned}$$

**Figure 17:**
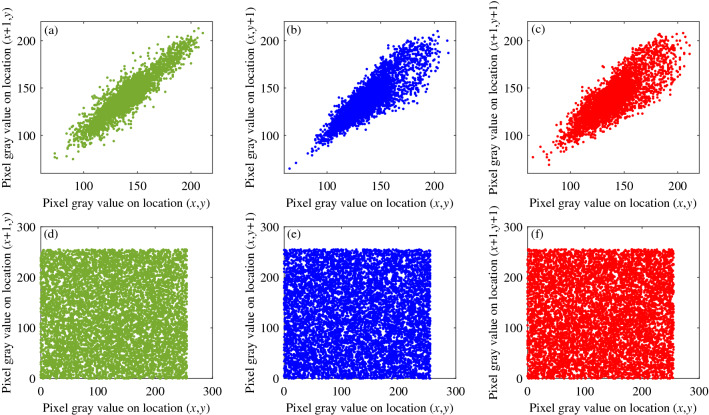
Correlation images of plaintext and ciphertext in various directions. (**a**) Correlation of plaintext in horizontal direction. (**b**) Correlation of plaintext in vertical direction. (**c**) Correlation of plaintext in Diagonal direction. (**d**) Correlation of ciphertext in horizontal direction. (**e**) Correlation of ciphertext in vertical direction. (**f**) Correlation of ciphertext in Diagonal direction.

Figure 17 shows the correlation between plaintext and ciphertext in various directions. 8000 pairs of adjacent pixels are selected from the horizontal, diagonal and vertical directions, and the correlation coefficients are shown in Table 10.

**Table 10:** Correlation coefficient analysis of different image correlation directions.

Image	Plain image			Cipher image		
	Horizontal	Vertical	Diagonal	Horizontal	Vertical	Diagonal
Brick Wall	0.8936	0.8380	0.7958	− 0.0321	− 0.0003	− 0.0027
Sand	0.7215	0.7439	0.5500	0.0037	− 0.0036	− 0.0104
Motion	0.9775	0.9928	0.9737	0.0004	− 0.0031	− 0.0152
Grass	0.8286	0.7119	0.5905	− 0.0052	0.0017	− 0.0090
Toy vehicle	0.9804	0.9919	0.9745	0.0035	− 0.0075	− 0.0005

Tables 10 and 11 show the correlation in each direction of the plaintext that the correlation coefficient value of the plaintext is greater than 0.9, and the pixel-intensive points in the image are all near the diagonal; this proves that the correlation between adjacent pixels of the chosen plaintext image is powerful. After the encryption system is processed, the correlation coefficient values of the ciphertext image in all directions are close to 0. The pixel dense points in the image show irregular scattered distribution, indicating that the correlation of ciphertext image is weak; this proves that the system has good encryption performance.

**Table 11:** Comparative analysis of adjacent pixel dependencies.

Directions	Brick wall	Ref.^54^	Ref.^55^	Ref.^56^	Ref.^57^
Horizontal	− 0.0321	− 0.0084	0.0018	− 0.0005	0.0021
Vertical	− 0.0003	0.0041	0.0040	0.0029	0.0008
Diagonal	− 0.0027	− 0.0463	− 0.0006	0.0030	0.0005

Table 11 compares the correlation of adjacent pixels of Brick Wall image in different directions with the performance of classic images in other literatures, and observes that the correlation of adjacent pixels in different directions of ciphertext images is close to 0, which further shows that the chaotic encryption system proposed in this paper has positive encryption and decryption effects.

## Conclusion

This study proposes a new improved chaotic system and successfully analyzes the effect of varying the number of unknown parameters in the new system. The dynamic behavior change of a chaotic system caused by the exponential change of a single-state variable in the nonlinear term of the new system is compared and analyzed. The results indicate that when the index value range is close to positive infinity, the chaotic system may possibly have a chaotic attractor. Simulation analysis of the new system under different initial conditions are conducted through circuit simulation. Finally, the new system is successfully applied to an image encryption system, and an excellent encryption effect is achieved.

## Data Availability

The data that support the findings of this study are available within the article. Further requests can be made to the corresponding author.
